# Genome-Wide Characterization, Comparative Analysis, and Expression Profiling of *SWEET* Genes Family in Four *Cymbidium* Species (Orchidaceae)

**DOI:** 10.3390/ijms26093946

**Published:** 2025-04-22

**Authors:** Yonglu Wei, Jie Li, Jianpeng Jin, Jie Gao, Qi Xie, Chuqiao Lu, Genfa Zhu, Fengxi Yang

**Affiliations:** 1 Guangdong Key Laboratory of Ornamental Plant Germplasm Innovation and Utilization, Environmental Horticulture Research Institute, Guangdong Academy of Agricultural Sciences, Guangzhou 510640, China; weiyonglu@gdaas.cn (Y.W.); lijie@gdaas.cn (J.L.); jinjianpeng@gdaas.cn (J.J.); gaojie@gdaas.cn (J.G.); xieqi@gdaas.cn (Q.X.); luchuqiao@gdaas.cn (C.L.); zhugenfa@gdaas.cn (G.Z.); 2 School of Landscape Architecture, Beijing Forestry University, Beijing 100083, China

**Keywords:** orchid, *SWEET*, flower development, transcriptional regulatory

## Abstract

The SWEET (Sugar Will Eventually be Exported Transporters) protein family plays a key role in plant growth, adaptation, and stress responses by facilitating soluble sugar transport. However, their functions in *Cymbidium* remain poorly understood. This study identified 59 *SWEET* genes across four *Cymbidium* species, encoding conserved MtN3/saliva domains. Despite variations in exon-intron structures, gene motifs and domains were highly conserved. Phylogenetic analysis grouped 95 SWEET proteins from six species into four clades, with gene expansion driven by whole-genome, segmental, and tandem duplications. Cis-element analysis and expression profiling across 72 samples revealed diverse regulatory patterns. Notably, *SWEET* genes showed peak expression in floral development, leaf morph variations, and diurnal rhythms. qRT-PCR and transcription factor binding analysis further highlighted their regulatory roles in floral patterning, leaf variation, and metabolic rhythms. These findings provide a foundation for future studies on *SWEET* gene function and their potential molecular breeding value in orchids.

## 1. Introduction

Carbohydrates are vital biomolecules in plants, serving as key energy sources and storage compounds. They play essential roles in plant growth, development, and adaptation to both biotic and abiotic stresses [1]. Moreover, sugars significantly influence the economic value of various crops. Consequently, the composition and balance of fructose and glucose in storage organs can greatly affect the flavor and quality of crops such as grapes [2], tomato fruit [3], potato tubers [4], carrot roots [1], and many other flowering plants [5,6]. Plants are known to harbor three distinct families of sugar transporters: sucrose transporters (SUTs), monosaccharide transporters (MSTs), and the SWEET (Sugar Will Eventually Be Exported Transporters) proteins [7,8]. While MSTs and SUTs belong to the major facilitator superfamily, SWEET proteins represent a unique class with seven transmembrane-spanning domains [9]. Phylogenetic analysis has classified plant SWEET proteins into four clades (I, II, III, and IV) [10,11]. While SWEET proteins within the same clade share structural similarities, they often localize to distinct cellular compartments and perform various physiological functions, though they typically transport similar substrates [12]. For instance, in Arabidopsis, Clade I and Clade II SWEETs are involved in hexose transport, Clade III transports sucrose, and Clade IV facilitates hexose transport to the tonoplast [9,13]. These transporters are integral to plant growth, development, and defense responses, enabling long-distance sugar transport from source organs (e.g., leaves) to sink organs (e.g., seeds and flowers) [14].

The first members of the SWEET family, MtN3 and Saliva, were identified in Medi-cago truncatula and *Drosophila melanogaster* in the late 1990s [15,16]. SWEET proteins are characterized by the conserved MtN3_saliva (MtN3_slv) transmembrane helix domains (TMHs), with their N-terminal facing externally and the C-terminal positioned towards the cytoplasmic membrane [17,18]. These proteins generally exhibit significant structural homology across model plants and crops, featuring seven TMHs and a 3-1-3 structure formed by two MtN3_slv domains [10]. To date, *SWEET* genes have been identified in a variety of plants, including 6 in moss (*Physcomitrella patens*) [10], 17 in *A. thaliana* (*Arabidopsis thaliana*) [10], 21 in rice (*Oryza sativa*) [11], 23 in *Bletilla striata* [19], 24 in maize (*Zea mays*) [20,21], 25 in *Dendrobium officinale* and rose (*Rosa chinensis*) [22,23], 52 in soybean (*Glycine max*) [24], 55 in *Gossypium hirsutum* [25], 77 in Strawberry (*Fragaria* × *ananassa Camarosa*) [26], and 108 in wheat (*Triticum aestivum*) [27], suggesting a considerable expansion of this family across both monocot and dicot species [28]. SWEET family members display distinct spatiotemporal expression patterns, which are linked to variations in sugar transport across different tissues [13].

The rich genetic diversity of Cymbidium makes it an ideal model for studying novel gene functions [29,30]. To date, full genomes of four *Cymbidium* species have been sequenced: *Cymbidium sinense* (*C. sinense*) [31], *Cymbidium ensifolium* (*C. ensifolium*) [32], *Cymbidium goeringii* (*C. goeringii*) [33,34] and *Cymbidium mannii* (*C. mannii*) [35]. *C. sinense* is known for its diverse floral patterns and fragrance, earning the title “King of Fragrance” [36,37]. *C. ensifolium* is smaller with thick, color-changing leaves [38,39]. *C. goeringii* is distinguished by its elegant flowers, pleasant fragrance, attractive foliage, and cold tolerance [40,41,42]. *C. mannii* is an epiphytic orchid highly valued for its ornamental and medicinal properties [43,44]. To explore the potential novel roles of *SWEET* genes in the growth and development of *Cymbidium*, we identified 59 *SWEET* genes and conducted a thorough analysis of their gene structures, conserved motifs, phylogenetic relationships, chromosomal distribution, collinearity, and cis-acting element predictions. In addition, we investigated the expression profiles and possible regulatory mechanisms of these genes across four *Cymbidium* species. This research offers essential genetic insights and provides a solid theoretical foundation for advancing molecular breeding strategies for orchids, as well as for experimentally validating new functions of *SWEET* genes.

## 2. Results

### 2.1. Identification and Characterization of SWEET Genes in Four Cymbidium Species Genomes

**Figure 1:**
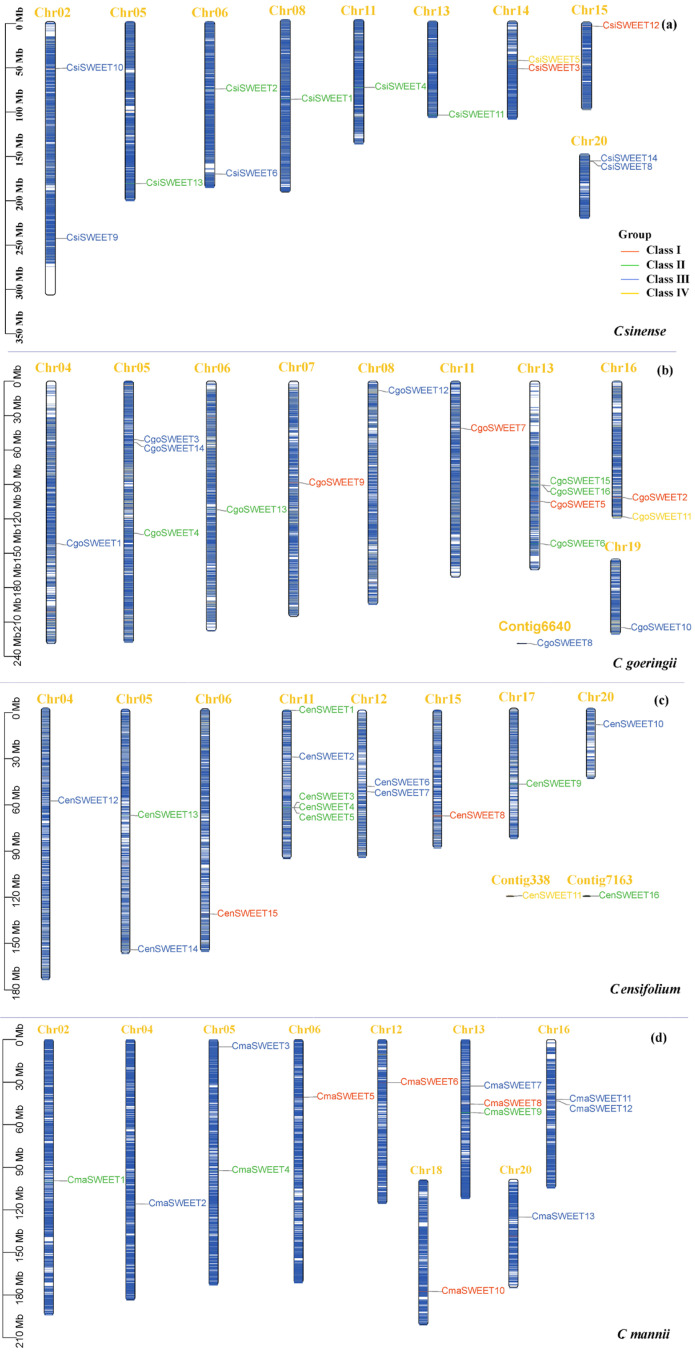
Chromosomal distribution of *SWEET* genes across four *Cymbidium* species. Chromosome names (indicated in yellow) are placed at the top, with gene names displayed on the right. The scale on the left represents distances in megabases (Mb). The heatmap gradient, ranging from red (high gene density) to blue (low gene density), illustrates the gene distribution on *Cymbidium* chromosomes, with an estimated inheritance interval of 300 kb. (**a**) *C. sinense*; (**b**) *C. goeringii*; (**c**) *C. ensifolium*; (**d**) *C. mannii*.

A total of 13, 14, 16, and 16 *SWEET* genes were identified in the genomes of *C. manniis*, *C. sinense*, *C. goeringii*, and *C. ensifolium*, respectively. These genes were designated as *CmaSWEET 1*-*13*, *CsiSWEET 1*-*14*, *CgoSWEET 1*-*16*, and *CenSWEET 1*-*16* based on their chromosomal locations. The coding sequences (CDS) of these *SWEET* genes ranged from 642 bp (*CsiSWEET14*) to 1419 bp (*CmaSWEET9*), while the corresponding DNA sequence lengths varied from 1133 bp (*CenSWEET13*) to 66,428 bp (*CmaSWEET9*). Protein sizes ranged from 213 amino acids (CsiSWEET14) to 472 amino acids (CmaSWEET9), with molecular weights (MW) spanning from 23.96 kDa to 52.10 kDa. The isoelectric points (pI) of the proteins ranged from 6.94 (CenSWEET3) to 9.88 (CsiSWEET17). Thirty-seven of the identified CymSWEETs exhibited stability, as evidenced by an instability index of less than 40. The grand average hydropathicity values of CymSWEETs ranged from 0.27 to 1.07. The detailed physicochemical properties of the *SWEET* genes and proteins from the four species are summarized in Table S1. Chromosomal localization analysis revealed that the *SWEET* genes were unevenly distributed across the chromosomes of the four species, with the genes spread across 8, 9, 9, and 9 chromosomes in *C. ensifolium*, *C. goeringii*, *C. mannii*, and *C. sinense*, respectively (Figure 1 and Table S1). Notably, several chromosomes, such as Chr13 in *C. goeringii* and Chr11 in *C. ensifolium*, contained four or more *SWEET* genes, highlighting a region of gene clustering (Figure 1).

### 2.2. Phylogenetic Analysis of SWEET Family

**Figure 2:**
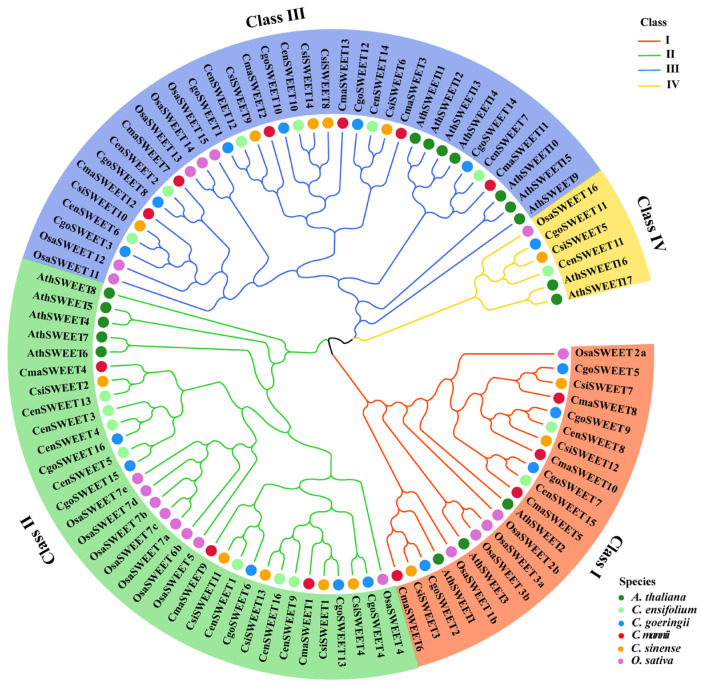
Phylogenetic tree showing the evolutionary relationships of SWEET proteins from four *Cymbidium* species (Cen, *Cymbidium ensifolium*; Cgo, *Cymbidium goeringii*; Cma, *Cymbidium mannii*; Csi, *Cymbidium sinense*), *Arabidopsis thaliana* (Ath), and *Oryza sativa* (Osa).

Phylogenetic analysis of 97 SWEET proteins revealed that they could be classified into four distinct groups: Clades I to IV. Clade III was the largest, consisting of 23 members, including 6 from *C. ensifolium* (*CenSWEETs*), 6 from *C. goeringii* (*CgoSWEETs*), 6 from *C. mannii* (*CmaSWEETs*), and 5 from *C. sinense* (*CsiSWEETs*). Clade II included 20 members, comprising 7 *CenSWEETs*, 5 *CgoSWEETs*, 5 *CsiSWEETs*, and 3 *CmaSWEETs*. Clade I contained 13 *SWEET* members, distributed as 2 in *C. ensifolium*, 3 in *C. sinense*, 4 in *C. mannii*, and 4 in *C. goeringii*. Clade IV, the smallest group, consisted of only one member per *Cymbidium* species, except for *C. mannii*. The phylogenetic tree indicated a mixed grouping pattern of the *SWEET* genes, rather than species-specific clustering, suggesting a complex evolutionary history of these transporters across different *Cymbidium* species (Figure 2).

### 2.3. Gene Structure, Protein Motif, and Domains of SWEET Members in Cymbidium

**Figure 3:**
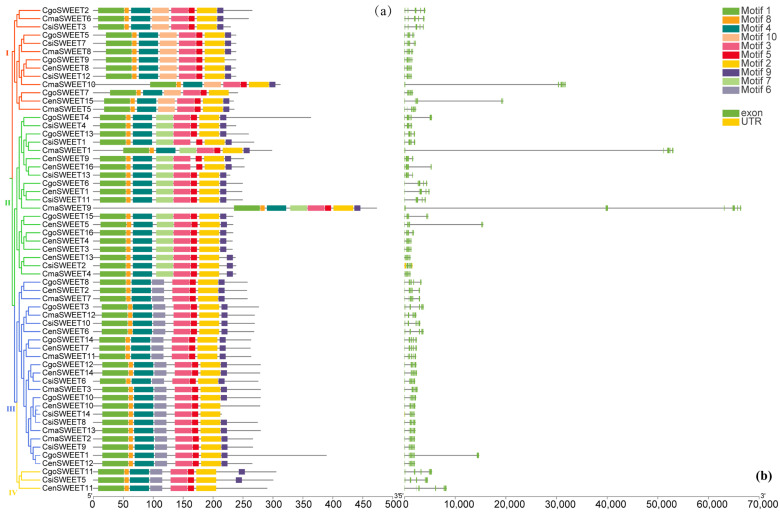
Phylogenetic relationships, conserved motifs, and gene structures of the *SWEET* gene family in four *Cymbidium* species. (**a**) Conservation of motifs within the *CymSWEET* gene family; (**b**) Gene structural variations of the *CymSWEET* family.

Motifs, which are conserved regions of protein or nucleotide sequences, play a critical role in regulating the function of genes and proteins through transcriptional and post-translational interactions. Among these, motifs 1, 2, 3, 4, 5, and 8 were universally present in all 59 CymSWEETs (Table S2). The next most prevalent motif, motif 9, was found in 54 of the CymSWEET proteins, excluding *C. ensifolium* (CenSWEET9 and CenSWEET10) and *C. goeringii* (CgoSWEET7 and CgoSWEET9). On the other hand, motifs 6, 7, and 10 were restricted to specific clades: motif 6 was absent in Clades I and II, motif 7 was unique to Clade II, and motif 10 appeared exclusively in Clade I (Figure 3a). The gene structure analysis revealed variation in exon numbers among the *CymSWEETs*, ranging from three (e.g., *CgoSWEET9*, *CgoSWEET11*, *CgoSWEET13*, and *CgoSWEET15*) to seven exons (e.g., *CmaSWEET9*, *CmaSWEET10*, and *CenSWEET11*). Interestingly, certain genes such as *CgoSWEET3*, *CenSWEET9*, *CenSWEET16*, *CsiSWEET1*, *CsiSWEET4*, and *CsiSWEET13* had four exons. The most common exon counts were five (13 instances, 22.03%) and six (33 instances, 55.93%) exons (Figure 3b and Table S1). In terms of transmembrane domains, all CymSWEET proteins except for CmaSWEET9 and CmaSWEET13 (which contain 8 transmembrane segments) possessed the typical seven transmembrane domains, consistent with the canonical structure of the SWEET family (Table S3). This finding highlights the conserved transmembrane architecture across the *CymSWEET* genes, which is fundamental to their function as sugar transporters.

**Figure 4:**
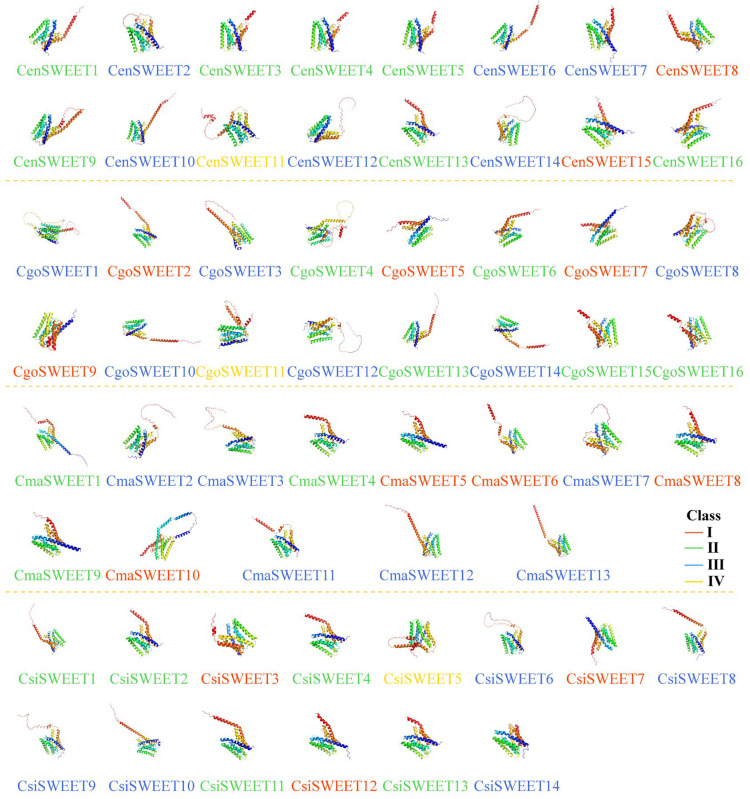
Tertiary structure models of SWEET proteins from the 59 identified *SWEET* genes across four *Cymbidium* species.

Furthermore, predictions of secondary protein structures highlighted that CymSWEET proteins predominantly comprised alpha helices (Hh) (35.08–74.79%), random coils (Cc) (30.17–51.10%), and extended strands (Ee) (12.92–19.74%) (Table S1). The 3D structures of CymSWEET proteins generated using AlphaFold2 at ColabFold [45] exhibited overall high similarity (Figure 4), yet notable distinctions were observed in their C-terminal regions. For example, the predicted structures indicated an additional α-helix in the C-terminal region of CsiSWEET8, absent in CsiSWEET14. Several studies underscore the functional significance of C-terminal regions in various transporters [46,47], suggesting that differences in these regions may impact sugar transport mechanisms.

### 2.4. Duplication and Collinearity of SWEET Genes in Cymbidium

**Figure 5:**
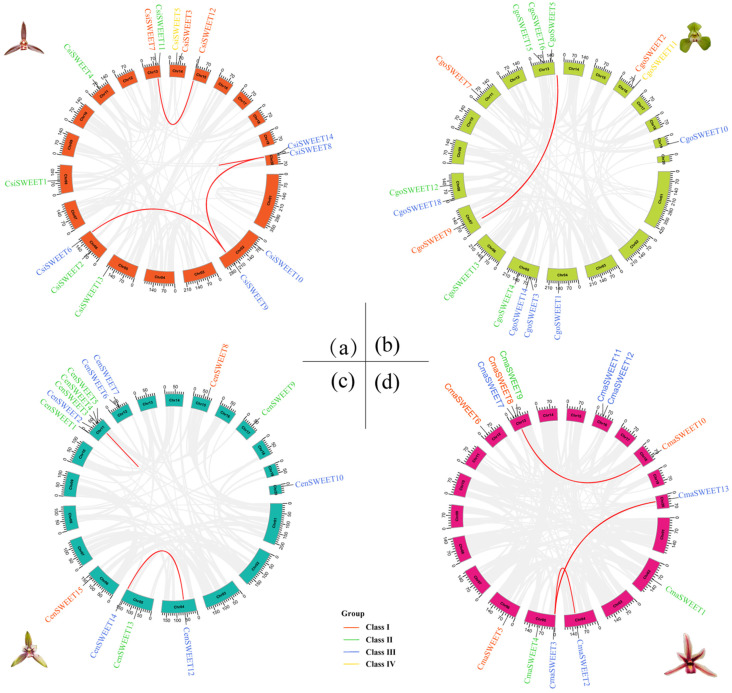
Chromosomal locations and gene duplication events of *SWEET* genes in the four *Cymbidium* species. (**a**) *C. sinense*; (**b**) *C. goeringii*; (**c**) *C. ensifolium*; (**d**) *C. mannii*. Red lines indicate the duplication events observed between genes.

To gain deeper insights into the phylogenetic dynamics of the Cymbidium *SWEET* gene family, we examined gene duplication events, which are crucial for understanding the evolutionary history and functional diversification of gene families. Gene duplications, whether tandem or segmental, play a key role in enhancing genetic diversity and driving adaptive evolution within species. Our analysis revealed segmental duplications in the genomes of *C. goeringii*, *C. ensifolium*, *C. mannii*, and *C. sinense*, with varying numbers of duplicated gene pairs: *C. goeringii* (*CgoSWEET5* and *CgoSWEET9*), *C. ensifolium* (*CenSWEET12* and *CenSWEET14*), *C. mannii* (*CmaSWEET2* and *CmaSWEET3*), and *C. sinense* (*CsiSWEET6* and *CsiSWEET9*). In addition, *C. ensifolium* exhibited one pair of tandem duplications (*CenSWEET3* and *CenSWEET4*). To further investigate the evolutionary pressure on these duplicated genes, we calculated the Ka/Ks ratios (the ratio of non-synonymous to synonymous substitution rates), which provide an indication of the selection pressure acting on the duplicated gene pairs. The Ka/Ks ratios for the 10 duplicated *SWEET* gene pairs in the Cymbidium genomes were consistently below one, suggesting that purifying selection is acting on these genes, maintaining their functional integrity over time (Figure 5 and Table S4). This observation supports the idea that the duplicated *SWEET* genes are functionally conserved and may have undergone evolutionary processes that retain their essential roles in the plant’s sugar transport system.

**Figure 6:**
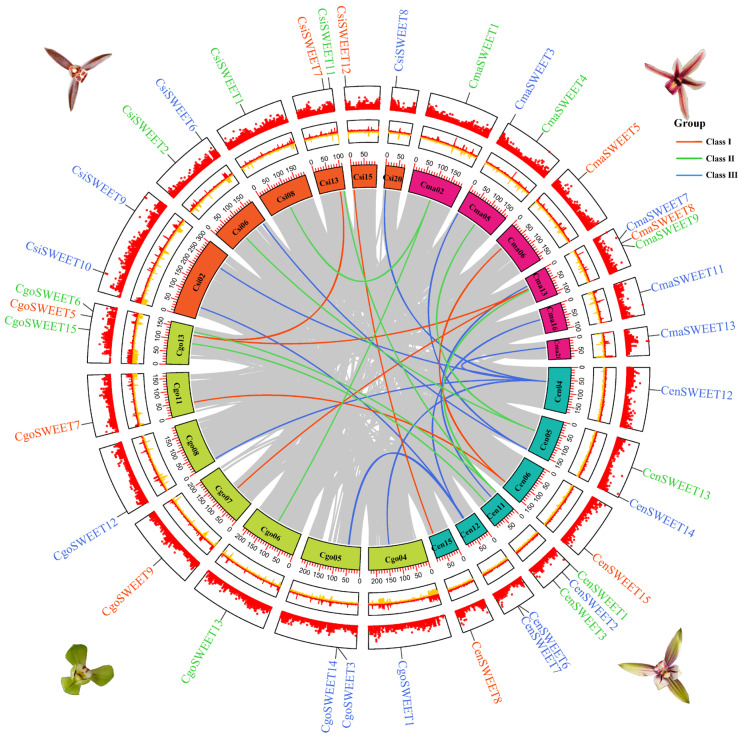
Homologous gene pairs within the SWEET family across four *Cymbidium* species (Cen, *Cymbidium ensifolium*; Cgo, *Cymbidium goeringii*; Cma, *Cymbidium mannii*; Csi, *Cymbidium sinense*). The concentric circles represent gene density, GC content deviation from the average, pseudochromosome positioning, and gene duplication. In-paralog pairs of *CymSWEET* genes are linked by color-coded lines in the innermost circle.

To further investigate the evolutionary relationships and structural organization of *SWEET* genes within *Cymbidium*, we performed synteny analysis across three species. This analysis identified 35 *SWEET* genes that were anchored within syntenic blocks, revealing conserved genomic regions across species. Intragenomic comparisons within *Cymbidium* highlighted 27 collinear gene pairs, demonstrating a significant degree of gene conservation and organization within the genome (Figure 6 and Table S5). Some genes were involved in multiple collinear pairs, such as *CenSWEET12*, which formed pairs with *CgoSWEET1*, *CgoSWEET12*, *CmaSWEET13*, *CsiSWEET8*, and *CsiSWEET9*, suggesting these genes have maintained functional and structural conservation across species. Intergenomic synteny comparisons further revealed 8 orthologous gene pairs between *C. sinense* and *C. ensifolium*, 7 between *C. ensifolium* and *C. goeringii*, 7 between *C. ensifolium* and *C. mannii*, 3 between *C. goeringii* and *C. mannii*, and 1 orthologous gene pair each between *C. sinense* and *C. goeringii*, and *C. sinense* and *C. mannii*. These orthologous gene pairs were distributed across different chromosomes, highlighting the dynamic nature of *SWEET* gene family duplications and the role they have played in shaping the evolutionary trajectory of all four *Cymbidium* species. Overall, these synteny findings emphasize the conserved nature of *SWEET* genes across different *Cymbidium* species and suggest that gene duplications, both within and across species, have been instrumental in the diversification and functional evolution of the *SWEET* gene family in these orchids.

### 2.5. Analysis of Cis-Acting Elements Analysis of SWEET Genes in Cymbidium

**Figure 7:**
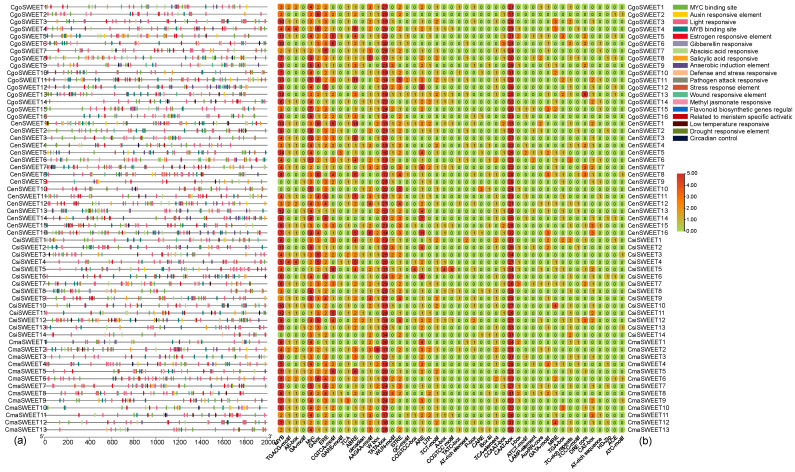
Identification of cis-regulatory elements within the promoter regions of *CymSWEET* genes. (**a**) Distribution of cis-acting elements across the 2000 bp upstream regions of the *CymSWEET* promoters; (**b**) Quantification of cis-acting elements present in the predicted promoter regions.

Our analysis revealed a rich array of cis-elements, with notable functional categories. Among them, thirty-three elements were responsive to light, fifteen were associated with hormone responses, and eight were linked to stress tolerance (Figure 7 and Table S6). Among the most widely conserved cis-elements across all 59 *CymSWEET* genes were the MYC motif, TATA-box, and CAAT-box, which were found in all genes. In addition, specific motifs such as the MYB and ERE motifs (ATTTTAAA, estrogen-responsive element) were present in 56 and 53 genes, respectively, indicating potential roles in transcriptional regulation related to environmental cues and phytohormone signaling. The most frequently observed cis-elements included the AuxRR-core (GGTCCAT), an aux-in-responsive element, which appeared 1589 times, and the TCT-motif (TCTTAC), a light-responsive element, which occurred 1285 times. These motifs suggest that *Cymbidium SWEET* genes are tightly regulated by light and auxin, two critical factors in plant growth and development. Furthermore, approximately 77.97% of the *SWEET* genes contained the anaerobic induction element ARE (AAACCA), which is crucial for responses to low oxygen conditions, and 45 genes harbored the stress-responsive STRE motif (AGGGG), known to be involved in stress adaptation. Another prominent feature was the ABA-responsive AAGAA motif (GAAAGAA), found in 44 *SWEET* genes, indicating potential involvement in abscisic acid-mediated stress responses. Interestingly, the conservation of cis-elements was evident within homologous *SWEET* gene pairs across different *Cymbidium* species. For example, the orthologous group consisting of *CenSWEET1*, *CgSWEET6*, *CsiSWEET11*, and *CmaSWEET9* displayed a particularly high number of MYB motifs, suggesting that these genes may share similar regulatory mechanisms across species. These findings highlight the complex and conserved regulatory network controlling *SWEET* gene expression in Cymbidium, with potential implications for their roles in plant growth, development, and responses to both environmental stresses and phytohormonal signals. The identification of these cis-elements provides valuable insights into the functional regulation of *SWEET* genes, paving the way for future research on their roles in orchid biology.

### 2.6. Expression Patterns of SWEET Genes in Cymbidium

**Figure 8:**
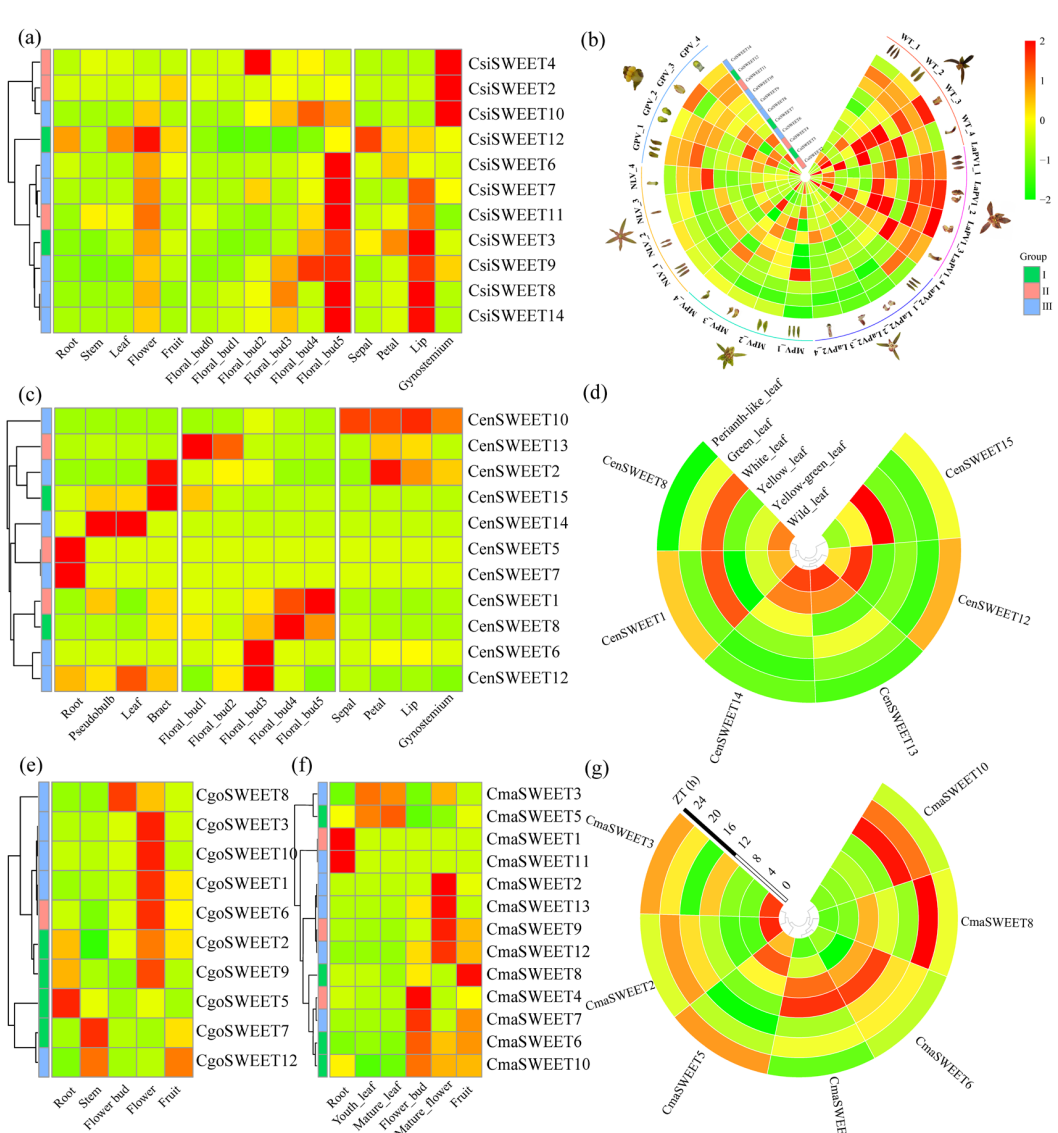
Expression patterns of *SWEET* genes in four *Cymbidium* species. (**a**,**b**) *C. sinense*. Floral bud0, dormant lateral buds; floral bud1, 1–5 mm floral bud; floral bud2, 6–10 mm floral bud; floral bud3, 11–15 mm floral bud; floral bud4, 16–20 mm floral bud; floral bud5, blooming flower. WT, wild type; LaPV, labellum-like perianth variety; MPV, multi-perianth variety; NLV, null-lip variety; GPV, gynostemium-like perianth variety. Numerals 1 to 4 denote specific floral organs: 1, sepal; 2, petal; 3, labellum; and 4, gynostemium. (**c**,**d**) Various tissues, organs, and leaf variants of *C. ensifolium*. (**e**) Different tissues in *C. goeringii*. (**f**,**g**) Tissue-specific and circadian analyses of *C. mannii*. Samples from 10 cm leaf-tip regions were collected every 4 h over a 24 h cycle, starting from 8:00 (ZT0, lights on) through 20:00 (ZT12, lights off), corresponding to time points ZT0, ZT4, ZT8, ZT12, ZT16, ZT20, and ZT24.

To gain deeper insights into the functional diversity of *Cymbidium SWEET* genes across various growth stages and tissues, we analyzed their expression patterns using transcriptome data from *C. sinense*, *C. ensifolium*, *C. goeringii,* and *C. mannii*. The heatmap in Figure 8 visualizes the FPKM (Fragments Per Kilobase of transcript per Million mapped reads) values of *SWEET* genes across multiple organs, including roots, stems, leaves, flowers, fruits, pseudobulbs, bracts, and various stages of floral development. Our analysis revealed that approximately 76.27% of the *Cymbidium SWEET* genes showed detectable expression (FPKM ≥ 1) in at least one tissue, while 14 out of the 59 genes exhibited no expression (FPKM < 1) across the tissues analyzed. Notably, certain *CsiSWEET genes* demonstrated robust expression (FPKM ≥ 30) across multiple tissues, including roots, stems, leaves, and different stages of floral development in *C. sinense*. These findings underscore the potential functional diversity of *CsiSWEET genes* across various tissues and developmental stages in *Cymbidium*.

Our analysis revealed that among the 14 *CsiSWEET* genes, nine (*CsiSWEET3*, *CsiSWEET6*, *CsiSWEET7*, *CsiSWEET8*, *CsiSWEET9*, *CsiSWEET10*, *CsiSWEET11*, *CsiSWEET12*, and *CsiSWEET14*) exhibit expression during floral development, suggesting that these genes may play important roles in the maturation of *Cymbidium* flowers (Figure 8a). Specifically, *CsiSWEET3* exhibited the highest expression in the lip, which is the nectar-secreting organ of orchids located at the base of the labellum. This suggests that *CsiSWEET3* may be involved in sugar unloading and transport within this critical tissue (Figure 8b). In addition, *CenSWEET14* showed unique expression patterns in both stem and leaf tissues (Figure 8c), with *CenSWEET1* predominantly expressed in white leaves and *CenSWEET12* showing the highest expression in yellow-green leaves (Figure 8d). These findings suggest that *CenSWEET* genes may be involved in the regulation of leaf and stem functions, potentially related to color development or metabolic processes. In the same vein, six *CgoSWEET* and eight *CmaSWEET* genes were highly expressed in the floral organs of *C. goeringii* (Figure 8e) and *C. mannii* (Figure 8f), respectively, indicating their potential involvement in floral development. Interestingly, *C. mannii*, valued for its medicinal properties, displays diurnal rhythmicity in metabolite accumulation [35,48]. Similarly, several *CmaSWEET* genes (*CmaSWEET5*, *CmaSWEET6*, *CmaSWEET8*, *CmaSWEET16*, *CmaSWEET19*, *CmaSWEET20*, and *CmaSWEET21*) exhibit diurnal fluctuations in expression levels (Figure 8g). This rhythmic expression suggests that these transporters play a key role in regulating plant metabolic processes, particularly in response to daily cycles. Overall, these expression data provide valuable insights into the functional roles of *SWEET* genes in *Cymbidium*, particularly their involvement in floral development, sugar transport, and metabolic regulation.

### 2.7. Evaluation of CsiSWEET Gene Expression Using qRT–PCR Analysis

**Figure 9:**
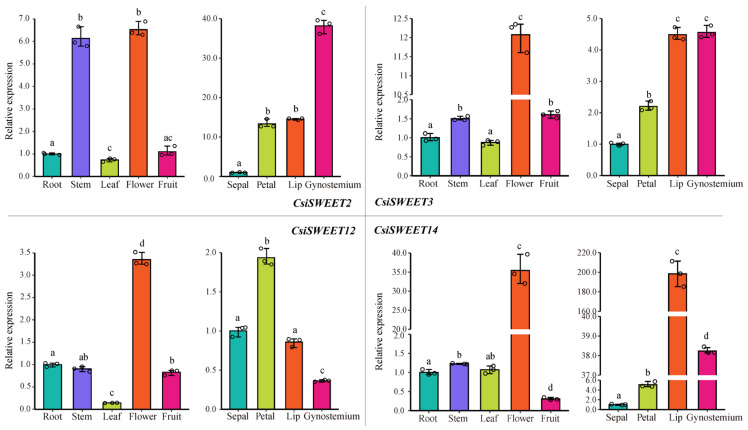
qRT-PCR analysis of the expression levels of four genes (*CsiSWEET2*, *CsiSWEET3*, *CsiSWEET12*, *CsiSWEET14*) in different tissues of *C. sinense*. Statistical significance was assessed using one-way analysis of variance (ANOVA), followed by Tukey’s post hoc test for multiple pairwise comparisons (Means with different lowercase letters differ significantly according to Tukey’s HSD post hoc test (*p* < 0.05)).

In order to conduct a more comprehensive analysis of the potential functions and to validate transcriptomic gene expression data pertaining to the *SWEET* gene family in various organs and tissues of Cymbidium, we specifically selected and evaluated four representative *CsiSWEET* genes that demonstrated the most significant differential expression in transcriptomic analyses and exhibited high expression levels in floral organs. Quantitative real-time PCR (qRT-PCR) analysis was subsequently performed for these four representative genes in roots, stems, leaves, flowers, fruits, and specific floral tissue parts (sepal, petal, lip, and gynostemium). The results were consistent with the transcriptome sequencing data, as shown in Figure 9. These four *CsiSWEET* genes exhibited distinct expression patterns across different tissues. For example, two class I genes (*CsiSWEET3* and *CsiSWEET12*) and one class III gene (*CsiSWEET14*) demonstrated particularly high expression in floral tissues, suggesting their significant roles in flower development. Specifically, *CsiSWEET14* showed substantial expression in the lip and gynostemium, areas critical for floral structure. The lack of an additional α-helix in its three-dimensional structure further hints at its functional specialization in these tissues. Additionally, *CsiSWEET2* exhibited notable expression in the gynostemium, suggesting its involvement in the formation of this reproductive organ. The qRT-PCR results closely matched the transcriptome sequencing data, reinforcing the idea that *CsiSWEET* genes play essential roles in floral tissue development. These findings also indicate that some of these genes are expressed in the stem, suggesting their potential broader functional roles in other parts of the plant as well.

## 3. Discussion

Sugars are essential for a wide range of plant life processes, which makes SWEET proteins, key players in sugar transport, particularly interesting. Unlike many transporters, SWEET proteins operate independently of environmental proton gradients, making them a unique focus of recent research [49,50]. While *Cymbidium* is one of the most diverse genera in orchids, its SWEET proteins have been relatively underexplored. This study represents the first comprehensive identification and comparative analysis of the *SWEET* gene family across four *Cymbidium* species at the whole-genome level. Our findings reveal that the *CymSWEET* genes form four distinct clades, a pattern that mirrors what has been observed in model plants like Arabidopsis and rice. However, we observed the absence of group IV members in *C. mannii*, which may be due to gene loss or replacement by functionally analogous genes [51,52]. Moreover, we identified one tandem and nine segmentally duplicated *CymSWEET* gene pairs across the *Cymbidium* genomes. All duplicated gene pairs displayed Ka/Ks ratios below one, indicating that purifying selection is acting on these genes to maintain their functional integrity over time. Such purifying selection is often associated with conserved biological processes critical for plant survival and fitness, including floral development, nectar secretion, and reproductive success. Previous studies in other orchid species such as *Dendrobium officinale* support this notion, highlighting similar patterns where *SWEET* gene duplications under purifying selection correlate with specific developmental and physiological roles, particularly in floral and stem tissues [22]. This highlights the significant role of gene duplication events in expanding and diversifying the *SWEET* gene family in *Cymbidium* [53,54]. The distinct expression patterns among duplicated *CymSWEET* genes further suggest that these genes have undergone functional divergence following duplication events. This divergence may play a critical role in the species-specific adaptations of Cymbidium to its ecological niche.

SWEET proteins typically feature a structure comprising seven α-helical transmembrane domains (TMs), which have been extensively studied across various species, revealing both conservation and diversification [21,55,56,57]. Our sequence analysis identified 52 candidate genes in *Cymbidium* containing the MtN3/saliva domain but fewer than seven TMs. This subset includes 11 candidates each from *C. goeringii* and *C. mannii*, and 15 from *C. goeringii* and *C. mannii*, which could play roles beyond sugar transport in processes such as organic acid, amino acid, or ion transport, regulation of signal transduction pathways, responses to environmental stresses, and intercellular interactions [58,59]. Moreover, the discovery of these homologous genes contributes to understanding the origins and evolutionary trajectories of *SWEET* genes.

Interestingly, the 14 *SWEET* genes with low or no expression (FPKM < 1) raise important questions about their potential biological relevance. These genes might represent pseudogenes that have lost their functionality due to mutations accumulated over evolutionary time. However, it is also plausible that these genes could be conditionally activated under specific environmental conditions or developmental cues not examined in our current studies, such as abiotic stresses (e.g., drought, salinity, temperature extremes), biotic stresses (e.g., pathogen attack), or symbiotic interactions [58,60,61]. Indeed, studies in *D. officinale* have shown that *SWEET* genes exhibit stress-responsive expression patterns, indicating their roles in plant adaptive responses under variable environmental conditions [22]. Therefore, additional experiments under various stress and symbiotic conditions would be valuable to clarify whether these lowly expressed *SWEET* genes in Cymbidium function conditionally or are truly pseudogenes.

While the role of SWEET proteins in sugar transport is well-established, our understanding of their regulatory pathways remains incomplete due to insufficiently identified regulators [17]. For example, in sugarcane, 20 genes binding to the *SsSWEET13c* promoter were identified via yeast one-hybrid (Y1H) assays, influencing leaf gradient development and circadian rhythms [62]. In soybeans, *GmST05* positively regulates seed size and affects seed oil and protein content by modulating GmSWEET10a transcription [63]. In rice, *OsDOF11* enhances resistance to sheath blight disease by binding to the *OsSWEET14* promoter, thereby activating its expression and reducing cellular sugar levels [64]. In Arabidopsis, under drought conditions, ABA activates the protein kinase AtSnRK2, which phosphorylates *AtSWEET11/12*, enhancing their oligomerization and sucrose transport activity to increase root sucrose content, thereby promoting root growth and enhancing drought resistance [65].

**Figure 10:**
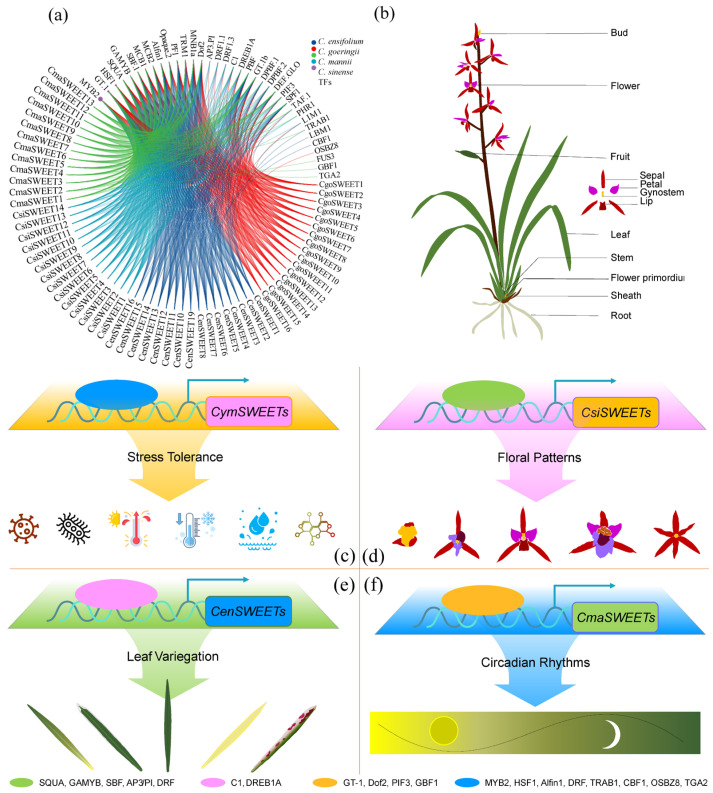
Predicted transcription factor binding sites within the promoter regions of *CymSWEET* genes and their potential regulatory functions. (**a**) Distribution of identified transcription factor binding sites (TFBSs) in the upstream promoter regions of *CymSWEET* genes; (**b**) Diagram of *Cymbidium* with the relevant regulatory regions highlighted; (**c**) Schematic model illustrating the potential transcriptional regulatory mechanisms involved in stress responses in *Cymbidium*; (**d**) Predicted regulatory network associated with floral patterning in *C. sinense*. (**e**) Proposed regulatory mechanisms underlying foliar variegation in *C. ensifolium*. (**f**) Putative regulatory framework related to circadian rhythm responses in *C. mannii*.

To explore potential upstream transcriptional regulation mechanisms of *SWEET* genes in *Cymbidium*, we employed computational predictions of transcription factor binding sites using PROMO [66]. This analysis identified 8460 binding sites across 37 types within the *CymSWEET* promoter regions (Figure 10a,b). Among these, MYB transcription factor MYB2, GT-1-like transcription factor (GT-1), and heat shock transcription factor 1 (HSF1) exhibited the highest number of binding sites, with 3562, 1318, and 1318 sites, respectively (Figure 10c, Table S7). MYB2 is involved in stress responses, modulating *SWEET* gene expression to enhance drought, cold, and salt tolerance by affecting sugar partitioning [67,68]. GT-1 regulates light-responsive gene expression, influencing photosynthetic efficiency and sugar distribution via *SWEET* gene regulation [69,70]. *HSF1* helps plants respond to heat stress, potentially maintaining normal sugar metabolism by regulating *SWEET* genes under high-temperature conditions [71]. Additionally, the involvement of MADS-box proteins SQUAMOSA (SQUA) and APETALA3/PISTILLATA (AP3/PI) in floral architecture regulation in *C. sinense* suggests they may modulate *SWEET* genes involved in these processes [72,73] (Figure 10d). The C1 and dehydration-responsive element binding protein (DREB) family transcription factors, associated with anthocyanin biosynthesis and chlorophyll metabolism, may influence leaf variegation in *C. ensifolium* through *SWEET* gene regulation [74,75] (Figure 10e). Phytochrome-interacting factor 3 (PIF3) and G-box Binding Factor 1 (GBF1) are implicated in light signal responses, potentially regulating rhythmic metabolite synthesis in *C. mannii* by modulating *SWEET* genes [35,76,77] (Figure 10f). Of course, applying these well-supported inferences to the practical aspects of orchid molecular breeding still requires traditional experimental validation, such as gene editing, yeast two-hybrid assays, chromatin immunoprecipitation, gene silencing or overexpression, as well as stress and hormone treatments.

## 4. Materials and Methods

### 4.1. Plant Materials and Growth Conditions

Orchid plants (*C. sinensis* ‘Qihei’) were cultivated in a growth chamber (LRH-1500 C-GSIE, Taihongjun Instruments, Shaoguan, China) at the Institute of Environmental Horticulture, Guangdong Academy of Agricultural Sciences (Guangzhou, China). The growth chamber maintained artificial climate conditions with 80% relative humidity and a temperature of 25 °C during a 16-h day and 18 °C during an 8-h night cycle an LED light source was employed, and the irradiance was adjusted to 55,000 lux. During the growth period, watering should be done 1–2 times per week, and potting media (brick pieces: coco chips: leaf mold at a ratio of 1:1:1) should remain moist. Mature plants aged two years were used for sampling, which was performed in three biological repeats, including roots, stems, young leaves (about 4 mm around wide), opening flowers, and fruits (mature but not cracked), which were immediately preserved at −80 °C after rapid freezing in liquid nitrogen and stored for RNA extraction.

### 4.2. Identification of the SWEET Gene Family in Four Cymbidium Species

Genomic data for *C. sinense*, *C. ensifolium*, *C. goeringii*, and *C. mannii* were retrieved from the National Center for Biotechnology Information (NCBI) (PRJNA743748, PRJNA597234) and National Genomics Data Center (NGDC) BioProject (PRJCA005355, PRJCA006911). Arabidopsis SWEET protein sequences from TAIR (https://www.arabidopsis.org/, accessed on 22 October 2024) were used as queries for BLASTP searches (e-value ≤ 1 × 10^−5^) against annotated proteins to identify orchid SWEET homologs in the *Cymbidium* protein database. Additionally, the hidden Markov model (PF03083) of the *SWEET* gene domain was employed to identify SWEET proteins via HMMER 3.2.1 software, selecting hits with E-value < 0.001 [78,79]. Combining results from both methods enabled the identification of *Cymbidium* SWEET proteins, followed by analysis of predicted molecular weights and theoretical isoelectric points using ProtParam (https://web.expasy.org/protparam/, accessed on 26 October 2024) [80].

### 4.3. Homology, Collinearity, Synteny and Duplication Analysis

Orthologous genes among the four *Cymbidium* species were identified using Or-thoFinder with default parameters [81]. Whole proteome sequences of *C. sinense*, *C. ensifolium*, *C. goeringii*, and *C. mannii* were aligned using the BLASTp program (e-value < 10^−5^). Internal collinearity blocks within target proteomes were identified using MCScanX with default parameters [82]. Duplication pairs were identified based on coding sequence homology [83].

### 4.4. Selection Pressure, Divergence Time, and Ka/Ks Calculation

Synonymous (Ks) and non-synonymous (Ka) substitution rates were computed using Clustal X2.0 software with default parameters for aligned SWEET duplicated gene pairs of *C. sinense*, *C. en-sifolium*, *C. goeringii*, and *C. mannii* [84]. TBtools-II v2.086 was employed to calculate Ks and Ka values [85]. A Ka/Ks ratio equal to one indicates neutral selection, greater than one indicates positive selection, and less than one indicates negative selection [86].

### 4.5. Multiple Sequence Alignment and Phylogenetic Analysis

Query sequences from TAIR (http://www.arabidopsis.org/, accessed on 22 October 2024) and RGAP (http://rice.uga.edu/, accessed on 22 October 2024) databases were downloaded for Arabidopsis and rice SWEET proteins, respectively [10,11]. Full-length SWEET protein sequences from *A. thaliana*, *O. sativa*, *C. sinense*, *C. ensifolium*, *C. goeringii*, and *C. mannii* were aligned using MAFFT (default parameter: FFT-NS-2) [87]. Phylogenetic analysis was performed using IQ-TREE v.2.2.0-beta with the maximum likelihood (ML) algorithm with 1000 bootstrap replicates [88]. The resulting Newick file (NWK) was visualized using the EvolView platform, available at https://www.evolgenius.info/evolview-v3 (accessed on 7 November 2024) [89].

### 4.6. Conserved Motif, Gene Structure, and Promoter Region Analysis

MEME Suite Version 5.3.3 (https://meme-suite.org/meme/tools/meme, accessed on 23 October 2024) predicted conserved motifs of each identified CymSWEET protein, setting maximum motif number to 10, motif width to 6–50 amino acids, and using default parameters for residuals [90]. Gene structure data were extracted from the *Cymbidium* genome GFF file and visualized using TBtools-II v2.086 [85]. Secondary structures of CymSWEET proteins were predicted using the SOPMA server [91], and 3D (three-dimensional) molecular models were constructed using AlphaFold2 and visualized with Pymol [92,93]. Cis-regulatory elements within gene promoters (2000 bp before ATG) were analyzed using the PLACE database (http://www.dna.affrc.go.jp/PLACE/, accessed on 26 October 2024) [94], with potential TF-binding sites identified using PROMO v.3.0.2 and compared against the TRANSFAC database [66,95]. ECharts provided graphical visualization [96].

### 4.7. Expression Pattern Analysis of CymSWEET Genes

Transcriptome data for various Cymbidium tissues were obtained from NCBI BioPro-jects PRJNA743748 and PRJNA597234, and NGDC BioProjects PRJCA005355 and PRJCA006911. After preprocessing with trimmomatic 0.39 [97], clean reads were mapped to the respective genomes using HISAT2 [98]. RSEM (v1.3.0) normalized reads to FPKM (fragments per kilobase of transcript per million fragments mapped reads) values using RSEM (v1.3.0) [99], and expression changes were calculated as log_2_ (mean FPKM of experimental group/control group). Heatmaps were generated using the R package ‘pheatmap’ and TBtools-II v2.086 [85].

### 4.8. RNA Isolation and qRT-PCR Analysis

Total RNA extraction used the RNAprep Pure Plant Kit (TIANGEN, Beijing, China) with three biological replicates. Reverse transcription was conducted with TransScript One-Step gDNA Removal and cDNA Synthesis SuperMix (TRANS, Beijing, China), and qRT-PCR used SYBR Green I Master Mix (Takara, Dalian, China) with a Light Cycler 96 Real-Time PCR System (Roche, Basel, Switzerland). The thermal cycling conditions were as follows: initial denaturation at 95 °C for 10 min, followed by 40 cycles of 95 °C for 15 s (denaturation) and 60 °C for 1 min (annealing/extension). The β-actin gene (Mol013347) in *C. sinense* was used as an internal reference. Relative expression levels were calculated using the 2^−ΔΔCT^ method with three biological and three technical replicates [100]. Data analysis was conducted in R v.4.0. Statistical comparisons of gene expression between tissues were performed using one-way ANOVA, followed by Tukey’s HSD (Honestly Significant Difference) post hoc test for pairwise comparisons. A *p*-value < 0.05 was considered statistically significant. Primer 5.0 (http://www.premierbiosoft.com/, accessed on 26 December 2022) was used to design primers for qRT-PCR analysis. Primer sequences are listed in Table S8.

## 5. Conclusions

The identification of 59 *SWEET* genes across four *Cymbidium* species led to their classification into four distinct classes. Phylogenetic analysis revealed the absence of the Class IV subfamily in *C. mannii*. Notably, *C. ensifolium* exhibited the highest number of collinear genes among the species, with 7 pairs each shared with *C. goeringii* and *C. mannii*, and 8 pairs with *C. sinense*. The genomic distribution of the entire *CymSWEET* gene family was uneven across chromosomes, with significant variations in sequence characteristics and genetic structures among its members. Sequence repeat analysis identified *CenSWEET3* and *CenSWEET4* as tandem repeats, while the remaining 9 pairs were classified as segmental repeats across the four *Cymbidium* species. Expression profiling showed that 45 *CymSWEET* genes were actively expressed, predominantly in flowers and leaves, highlighting their potential significance in the diversity of orchid species. Additionally, the presence of various cis-acting elements, regulatory factors, and gene action studies emphasized the impact of genetic backgrounds, regulatory elements, stress types, and tissue-specific expression on the functional specialization and dominance of CymSWEET transporters. These findings provide a solid foundation for future in-depth investigations into the roles of *CymSWEET* genes in growth, mutation, and stress tolerance, and lay the groundwork for employing various functional validation approaches and genetic enhancements in orchids and other plant species.

## Data Availability

The raw data supporting the conclusions of this article will be made available by the authors upon request.

## Supplementary Materials

The following supporting information can be downloaded at https://www.mdpi.com/article/10.3390/ijms26093946/s1.
